# Disrupted cerebellar interactions in the cerebellar-thalamo-cortical triple network model across risk states and first-episode psychosis

**DOI:** 10.1038/s41537-026-00783-6

**Published:** 2026-07-16

**Authors:** Minji Ha, Inkyung Park, Hyungyou Park, Taekwan Kim, Wu Jeong Hwang, Jiseon Jang, Minah Kim, Jun Soo Kwon

**Affiliations:** 1 Institute of Human Behavioral Medicine, SNU-MRC Seoul, Republic of Korea; 2 Department of Psychiatry, Seoul National University College of Medicine, Seoul, Republic of Korea; 3 Department of Brain and Cognitive Sciences, Seoul National University College of Natural Sciences, Seoul, Republic of Korea; 4 Department of Bio and Brain Engineering, Information & Electronics Research Institute, Korea Advanced Institute of Science and Technology, Daejeon, Republic of Korea; 5 Department of Neuropsychiatry, Seoul National University Hospital, Seoul, Republic of Korea; 6 Department of Psychiatry, Hanyang University Hospital, Seoul, Republic of Korea

**Keywords:** Psychosis, Neural circuits

## Abstract

Cerebellar dysconnectivity has been repeatedly linked to psychosis and is often accompanied by disruptions in thalamic and cortical regions. These disturbances are broadly consistent with the triple network model, which conceptualizes psychopathology as arising from abnormal interactions among the salience (SAL), default mode (DMN), and executive control (ECN) networks. However, how the cerebellum interacts with thalamic and cortical components of these networks across different psychosis risk stages and early psychosis remains unclear. Resting-state functional MRI from 37 first-episode psychosis (FEP) patients, 63 clinical high-risk (CHR) individuals, 41 unaffected relatives (URs) of schizophrenia patients, and 100 healthy controls (HCs) were analyzed. The cerebellum and thalamus were parcellated according to their functional connectivity with cortical functional networks, and the DMN, SAL, and ECN subdivisions across all three regions were used to estimate cerebellar–cortical and cerebellar–thalamic connectivity across groups. Compared with HCs, FEP patients showed widespread increases in cerebellar–cortical connectivity across all three networks, together with reduced cerebellar–thalamic connectivity, most prominently within SAL-related circuits. CHR individuals exhibited predominantly increased connectivity, with localized disruptions across cerebellar, thalamic, and cortical regions. URs did not show significant connectivity differences relative to HCs. By extending the triple network framework to include cortico-thalamo-cerebellar pathways, this study characterized how cerebellar network connectivity differs across familial risk, CHR, and FEP groups. The observed pattern suggests that early changes in CTC circuits correspond to disruptions described within the triple network model, and highlights the potential relevance of cerebellar and thalamic involvement when characterizing network alterations across the psychosis spectrum.

## Introduction

The cerebellum has been shown to be associated with motor coordination and is now recognized as an important region for higher-order cognitive functions^1–3^. Previous neuroimaging studies have consistently revealed structural and functional abnormalities in the cerebellum across the psychosis spectrum^2,4–6^. These disturbances frequently co-occur with disruptions in thalamic and cortical networks, which have been observed not only in patients with schizophrenia^7–12^ but also in individuals at clinical high risk (CHR)^10,11,13^ and in patients experiencing first-episode psychosis (FEP)^9,10,12,14–16^. This pattern indicates that the cerebellar–thalamic–cortical (CTC) circuit is a central component of the neural dysfunctions characterizing psychotic disorders. Notably, similar though subtler patterns of cerebellar-cortical dysconnectivity have been found in unaffected siblings of schizophrenia patients, pointing to a possible genetic vulnerability^17–19^.

These insights appear consistent with several theoretical models that have been proposed to explain psychosis pathophysiology. The cognitive dysmetria theory^20^ suggests that connectivity disruptions across cortical, thalamic, and cerebellar regions may interfere with cognitive coordination in schizophrenia. Additionally, recent work has connected CTC dysfunction to predictive coding frameworks, proposing that impaired sensory prediction and error monitoring processes could contribute to the perceptual and cognitive features of psychotic disorders^21–23^. These findings also seem to align with network-level approaches to understanding psychosis, such as the triple network model focusing on interactions between the default mode (DMN), salience (SAL), and executive control (ECN) networks^24–28^. This framework suggests that altered SAL-mediated salience processing may disrupt the balance between internally oriented DMN activity and externally focused ECN control, thus contributing to cognitive and symptomatic difficulties. Evidence that thalamic and cerebellar subdivisions are represented by these intrinsic cortical networks^29–31^ further supports the notion that CTC dysconnectivity underlies pervasive network-level disturbances^26,32^. While these different theoretical perspectives have largely developed independently, the emerging pattern of CTC-related findings suggests they might offer complementary insights into the neural basis of psychosis when considered together.

The significance of these findings lies in reframing the brain as an intricately interconnected network wherein dynamic interactions underpin perceptual functions. We previously explored the premise of CTC dysconnectivity within the triple network model, revealing reduced connectivity across the integrative CTC network in schizophrenia patients^31^. However, reliance on a whole-mask approach provided only a coarse-grained perspective, thus obscuring the subtleties of these interactions. Moreover, by focusing exclusively on patients with chronic schizophrenia, we overlooked critical insights into connectivity disruptions during earlier stages of psychosis. To address these gaps, Kim et al^33^. examined thalamic-cortical connectivity within the triple network across FEP patients, CHR individuals, and genetically at-risk groups, thereby offering a more granular perspective on connectivity patterns within this large-scale framework across psychosis risk stages. However, their exclusion of the cerebellum has yielded an incomplete picture of CTC dysconnectivity within the triple network model.

Therefore, our approach explicitly incorporated cerebellar networks within each of DMN, SAL, and ECN and examined both cerebellar-cortical and cerebellar-thalamic connectivity patterns across psychosis risk states and FEP. We hypothesized that the FEP group would demonstrate the most pronounced network disconnections, thus reflecting the impact of full-blown psychotic episodes while also sharing interaction patterns with CHR. In contrast, the CHR group was expected to exhibit connectivity patterns distinct from those of the UR group, highlighting differences in vulnerability to psychosis across these populations. Given that CTC circuits have been proposed to play a role in predictive coding and error monitoring processes, we conducted an exploratory analysis to examine whether connectivity patterns within these networks might relate to cognitive performance. Specifically, we explored the relationship between cerebellar-thalamic and cerebellar-cortical connectivity and error-processing performance of the Spatial Working Memory (SWM) task in the FEP group.

## Methods and materials

### Demographic information

A total of 241 participants, including 37 FEP patients, 63 CHR individuals, 41 URs, and 100 HCs, were included in this study. FEP patients, CHR individuals, and URs were recruited from an inpatient and outpatient clinic of the Department of Neuropsychiatry and the Seoul Youth Clinic^34^ at Seoul National University Hospital (SNUH). The inclusion criteria for FEP patients were as follows: aged 16 to 40 years; met the diagnostic criteria for schizophreniform disorder, schizophrenia, or schizoaffective disorder according to the Structured Clinical Interview for the Diagnostic and Statistical Manual of Mental Disorders, Fourth Edition, Axis I Disorders (SCID-I); and a duration of psychotic illness of less than 2 years. Psychotic symptoms were assessed using the Positive and Negative Syndrome Scale (PANSS).

CHR individuals were assessed using the Structured Interview for Prodromal Symptoms^35^. To mitigate the influence of genetic predisposition, CHR individuals who had any relatives with a history of psychotic disorders or who met the criteria for schizotypal personality disorder were excluded from the study. URs were defined as individuals who did not exhibit any psychiatric symptoms and had no prior or existing diagnosis of any psychiatric disorder but had a family history of schizophrenia, such as one 1st-degree relative with a confirmed diagnosis of schizophrenia and one or more affected relatives within the 1st to 3rd-degree. The assessment of prodromal symptoms for both CHR and URs was conducted via the validated Korean version of the Scale of Prodromal Symptoms (SOPS)^36^.

HCs were recruited to SNUH through online advertisements. The SCID-I Nonpatient Edition was used to screen for Axis I disorders, and individuals with any family biological relatives within the 1st to 3rd-degree who had been diagnosed with a psychotic disorder were excluded from the study. The exclusion criteria were as follows: substance abuse or dependence (not including nicotine); neurological disorders or substantial head injuries; medical conditions potentially presenting with psychiatric symptoms; or intellectual disability (with an intelligence quotient (IQ) below 70). The IQ levels of all the subjects were evaluated using the Korean version of the Wechsler Adult Intelligence Scale.

Subsets of the data from this cohort have been used in other previous studies^16,33,37^. As cerebellar coverage was not a requirement in those prior studies, all 273 participants from the original dataset were initially considered for inclusion in the present study. Each participant’s resting-state fMRI scan was subsequently subjected to a systematic visual quality check to verify adequate cerebellar field-of-view coverage, given that cerebellar connectivity was central to the present research question. Based on this inspection, 32 participants (FEP = 2, CHR = 15, GHR = 5, NOR = 10) were excluded due to insufficient cerebellar coverage, yielding a final analytic sample of 241 participants (FEP = 37, CHR = 63, GHR = 41, NOR = 100).

All participants provided written informed consent after receiving a full explanation of study procedure in the previous prospective cohort study (IRB no. H-1110- 009-380). For minors, both the participants and their parents provided written informed consent. The authors assert that all procedures contributing to this work comply with the ethical standards of the relevant national and institutional committees on human experimentation and with the Helsinki Declaration of 1975, as revised in 2013. All procedures involving human participants were approved by the Institutional Review Board of SNUH (IRB no. H-2309-134-1470).

### Image acquisition

The MRI data were acquired using a 3 T Trio MRI scanner (Siemens Magnetom Trio, Erlangen, Germany) with a 12-channel head coil. Blood oxygenation level-dependent functional MRI images were acquired for 6 minutes and 58 seconds with a gradient echo planar imaging pulse sequence at echo time/repetition time (TE/TR) = 30/3500 ms, flip angle = 90°, field of view = 240 mm, matrix = 128 × 128, and voxel size = 1.9 × 1.9 × 3.5 mm^3^. While acquiring the resting-state fMRI data, the participants were instructed to keep their eyes closed. To reduce movement and stabilize the position, head cushions were used. In addition, T1 structural images were obtained with magnetization-prepared rapid gradient echo (MP-RAGE) at TE/TR = 1.89/1670 ms, flip angle = 9°, FOV = 250 mm, matrix = 256 × 256, and voxel size = 1.0 × 1.0 × 1.0 mm^3^.

### Preprocessing

Data preprocessing was performed via the CONN toolbox (version 19c), which is SPM-based software implemented in MATLAB 2021a^38^. The preprocessing steps included removal of the first four echo-planar images, realignment and unwarping, slice timing and motion correction, artifact detection tool-based outlier detection, automated brain extraction, T1-functional image registration, normalization to the Montreal Neurological Institute (MNI) space, and smoothing to a 6-mm full width at half maximum. To ensure motion quality control, we inspected the data and set the exclusion criteria of head motion parameters exceeding translation 2.0 mm and rotation >2.0°. The regression of nuisance signals from the white matter and cerebrospinal fluid was performed using aCompCor^39^ in addition to scrubbing, motion regression, linear detrending, and bandpass filtering at 0.008 < f < 0.09 Hz.

### Network parcellation

**Fig. 1:**
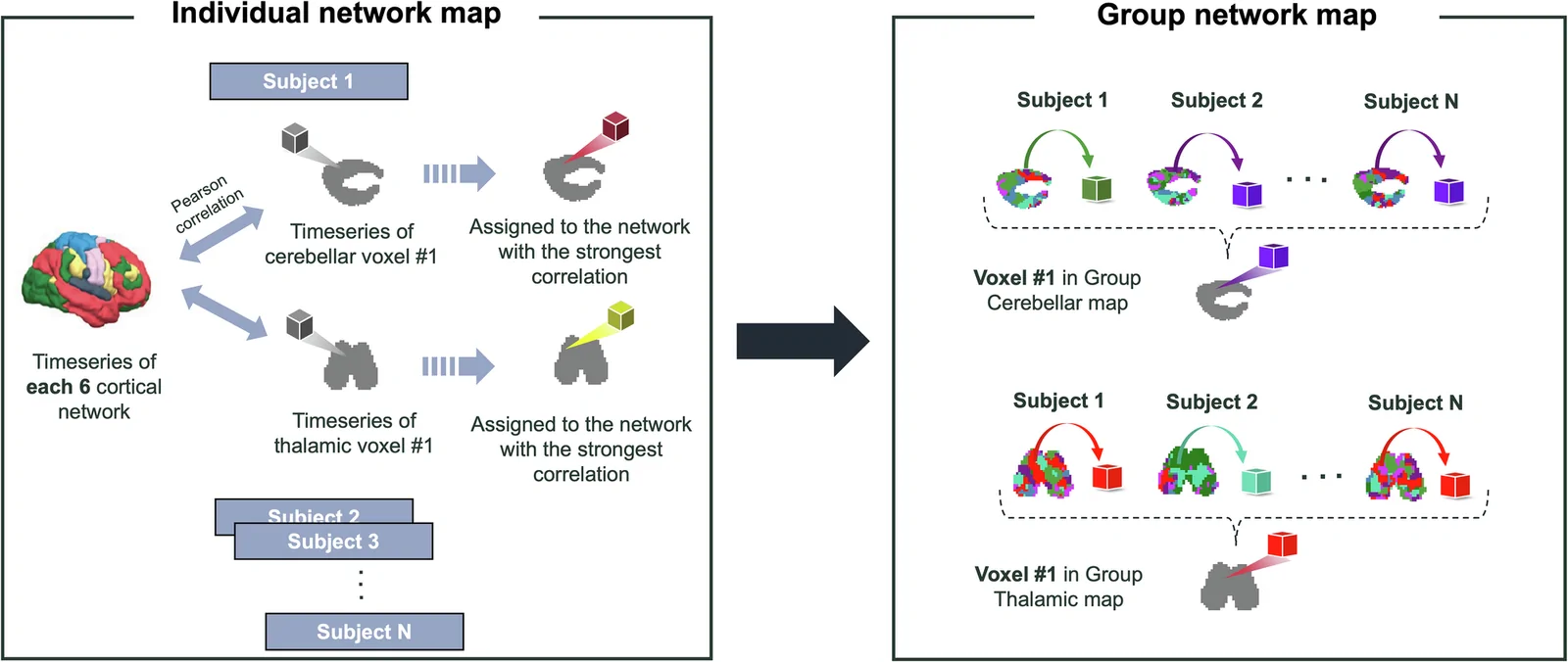
Visualization of the network parcellation process. The winner-take-all method was applied to the network parcellation of the thalamus and cerebellum. The Pearson correlation coefficients between the time series in each voxel of the cerebellar masks and the time series of each cortical network were computed, and each voxel was mapped to the network that had the highest positive correlation coefficient, i.e., the most relevant and related network.

For the cortical network parcellation map, we adopted the network map defined by Kim et al.^33^ on the same dataset. In Kim et al., cortical network modules were identified using the InfoMap community detection algorithm based on cortico-cortical functional connectivity matrices constructed from ROIs defined by Gordon et al (See Kim et al.^33^ for more details). Subject-level modular assignments were first estimated and subsequently averaged to generate a consensus group-level cortical network map^40,41^. In the present study, this previously established group-level map was used to define the cortical components of the triple networks (DMN, SAL, and ECN). For non-cortical structures, we utilized the thalamic parcellation map from the same study and independently generated a cerebellar parcellation, both employing the identical winner-take-all approach to map voxels to cortical networks^29–31,33,42^. For each participant, we calculated Pearson correlation coefficients between the time series of each cerebellar voxel and the average time series of each cortical network (Fig. 1). Cerebellar voxels were defined using the FSL MNI FNIRT cerebellum atlas. Each cerebellar voxel was then assigned to the cortical network that showed the highest positive correlation with that voxel’s timeseries. This process identified which of the three networks each cerebellar voxel was most functionally connected to. Finally, a group-level consensus map was created by determining which cortical network received the most frequent assignment for each cerebellar voxel across all participants, which ensured that cerebellar regions were assigned to networks based on consistent connectivity patterns across the sample.

### Analysis of functional network ROIs

The SAL, ECN, and DMN ROIs were separated from all three cortical, thalamic, and cerebellar network maps, resulting in a total of 9 network ROIs. A 6 × 6 functional connectivity matrix was created for each connectivity analysis: cerebellar‒cortical connectivity and cerebellar‒thalamic connectivity. Pearson’s correlation coefficients were used to evaluate the connectivity between each connection across all ROIs by transforming the coefficients into z scores via Fisher’s z-transformation. Group differences in ROI-to-ROI functional connectivity were assessed using the multivariate general linear model (GLM) framework implemented in the CONN toolbox. Statistical inference followed the Functional Network Connectivity cluster-level inference procedure implemented in CONN. In this framework, ROIs are organized into functional networks and clusters correspond to sets of ROI-to-ROI connections belonging to the same within- or between-network connectivity set. The multivariate GLM evaluates the entire set of connections between each pair of networks, which produces a cluster-level F-statistic that characterizes group differences in connectivity between the two networks. For network pairs showing a significant multivariate effect, individual ROI-to-ROI connections contributing to that effect are subsequently examined and reported using their connection-level statistics. In detail, connection-level statistics were first computed for each ROI-to-ROI pair. Connections exceeding the connection-level threshold (*p* < 0.05) were then grouped into clusters within the connectivity matrix, and for each cluster of suprathreshold connections a cluster-level statistic was computed. Cluster-level significance was assessed using false discovery rate (FDR) correction across clusters (*p* < 0.05). To compare CHR individuals and HCs, we used age as a covariate since there was a statistically significant group difference in age.

### Exploratory correlation analysis with error processing in FEP patients

Exploratory correlational analyses were performed to examine relationships between connectivity strength and cognitive performance in the FEP group, as this group represents the clinical onset stage where network disruptions would likely be most pronounced. We calculated Pearson correlations between functional connectivity values and error rates from the SWM task, which was administered outside of MRI scanning sessions. The SWM task is an error-based learning task from the Cambridge Neuropsychological Test Automated Battery (CANTAB), and measures error-based learning by requiring participants to locate hidden tokens within colored boxes while minimizing errors. In each trial, participants must remember which boxes they have already opened and avoid repeating selections, testing both spatial working memory and error monitoring abilities. All statistical analyses used two-sided tests.

## Results

### Participant characteristics

There were significant differences in age and IQ between groups. Post hoc analysis revealed that age was significantly lower in the CHR group than in the HC group (CHR vs. HCs, t = −5.66, *p* < 0.001). IQ was significantly lower in the FEP group than in the CHR (FEP vs. CHR, t = −3.83, *p* < 0.001), UR (FEP vs. URs, t = −2.85, *p* = 0.006), and HC groups (FEP vs. HCs, t = −5.76, *p* < 0.001). There were no differences in sex or handedness between groups. The PANSS scores of the FEP patients and the SOPS scores of the CHR individuals and URs are shown in Table 1.

**Table 1:** Demographic and clinical characteristics of all the subjects.

Variables	HCs (*n* = 100)	FEP (*n* = 37)	CHR (*n* = 63)	URs (*n* = 41)	Statistics		
					T/χ^2^/F	*p*	Post hoc
Demographic							
Age (years)	24.7 ± 6.7	23.1 ± 5.60	20.5 ± 3.2	23.0 ± 5.3	7.30	< 0.001	CHR < HCs^***^
Sex					7.49	0.058	
Male	60	15	41	25			
Female	40	22	19	16			
Handedness					6.70	0.46	
Right	94	32	54	39			
Left	6	5	5	2			
Both			1				
IQ	112.5 ± 12.3	98.5 ± 13.7	108.7 ± 12.8	107.2 ± 13.2	11.02	< 0.001	FEP < HCs^***^; FEP < CHR^**^; FEP < URs^*^
Clinical							
Antipsychotic Usage^a^		9.35 ± 7.74					
DOI (months)		5.84 ± 3.78					
DUP (months)		4.62 ± 3.93					
GAF	85.9 ± 4.2						
PANSS							
Total		68.5 ± 12.5					
Positive		16.8 ± 4.7					
Negative		16.9 ± 4.8			-		
General		34.9 ± 6.9					
SOPS							
Total			33.2 ± 12.1	3.9 ± 4.2			
Positive			9.6 ± 4.0	0.9 ± 1.4			
Negative			13.2 ± 6.7	1.5 ± 1.8			
Disorganized			3.8 ± 2.7	0.3 ± 0.5			
General			6.6 ± 4.4	1.2 ± 1.6			

*CHR* clinical high-risk, *DOI* duration of illness, *DUP* duration of untreated psychosis, *FEP* first-episode psychosis, *GAF* global assessment of functioning scale, *HCs* healthy controls, IQ intelligence quotient, *PANSS* positive and negative syndrome scale, *SOPS* scale of prodromal symptoms, *URs* unaffected relatives, *OZP-eq* olanzapine-equivalent.

^*^p *<* 0.05; ^**^p *<* 0.01; ^***^p *<* 0.001

Independent t-tests for continuous variables and chi-square tests for categorical data were performed.

^a^Olanzapine equivalent dose (mg) of antipsychotics.

### Cerebellar network parcellation

**Fig. 2:**
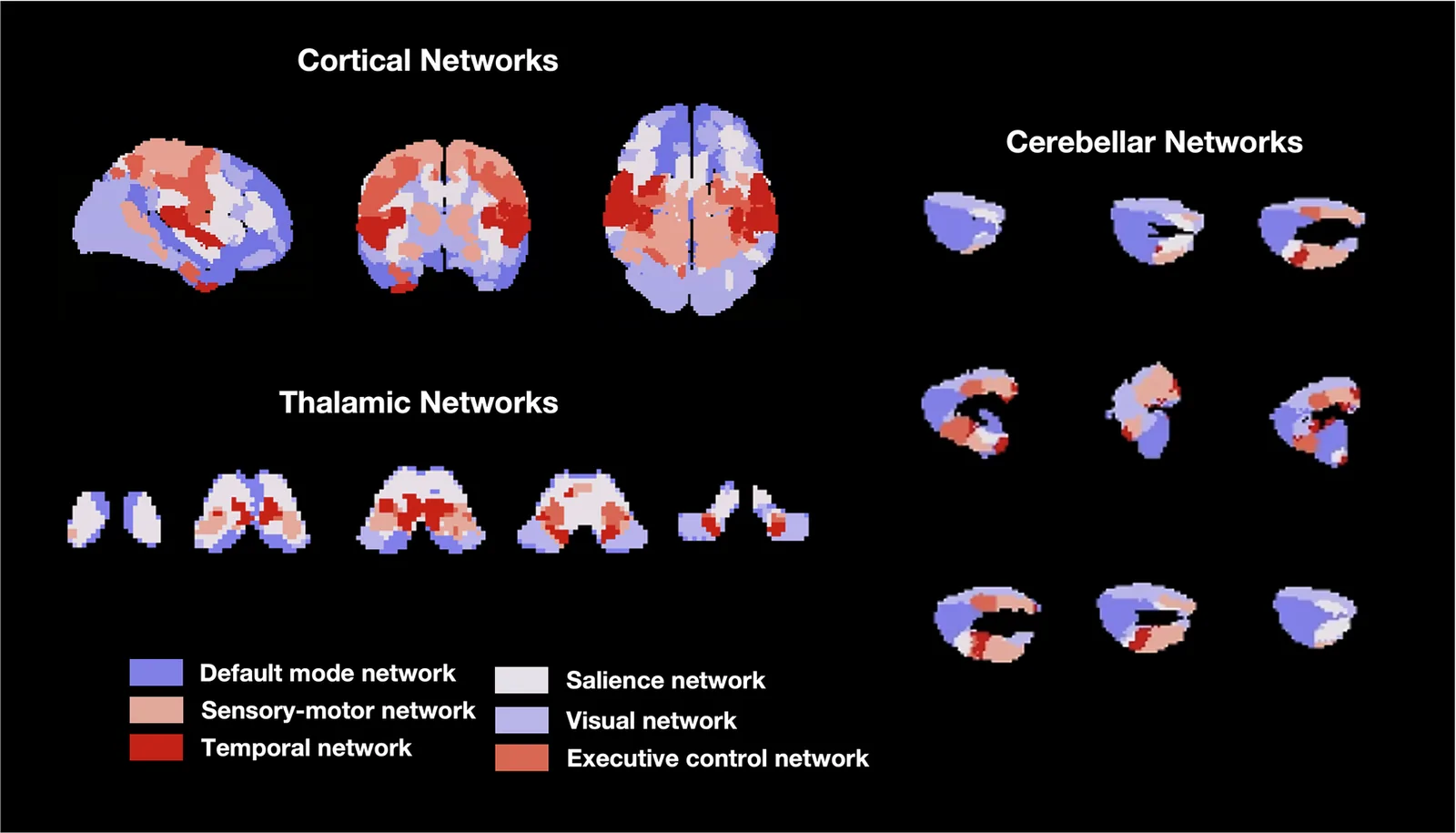
Network parcellations of the cortex, thalamus, and cerebellum. The cortical network maps were generated based on the modular organization. Thalamus and cerebellum were represented by the cortical networks via winner-take-all method.

Networks related to lower-level functions, such as visual and sensory-motor networks, were mapped to the anterior cerebellum, a region traditionally linked with motor control and basic sensory processing. In contrast, networks associated with higher-level cognition, such as the DMN, ECN, and SAL, were represented predominantly in the posterior cerebellum. This anterior‒posterior gradient aligns with established functional divisions in the cerebellum, with anterior regions supporting motor and sensory integration, whereas posterior regions connect with cortical areas responsible for complex cognitive processes^3,30^. The cerebellar, cortical, and thalamic network maps are shown in Fig. 2.

### Between-network connectivity: cortical–cerebellar network connectivity

Compared with HCs, the functional connectivity of cerebellar SAL with cortical DMN (*p* = 0.041, T = 2.06), cortical SAL (*p* = 0.038, T = 2.09), and cortical ECN (*p* = 0.006, T = 2.78) was greater in FEP patients, whereas the functional connectivity between cerebellar SAL and cerebellar DMN (*p* = 0.003, T = -3.00) was lower in FEP patients. FEP also revealed hyperconnectivity of cortical SAL with all three cerebellar networks (cortical SAL and cerebellar DMN, *p* = 0.030, T = 2.20; cortical SAL and cerebellar SAL, *p* = 0.038, T = 2.09; cortical SAL and cerebellar ECN, *p* = 0.008, T = 2.69). In contrast to the comparison between FEP patients and HCs, CHR individuals exhibited hyperconnectivity between cerebellar SAL and cerebellar ECN (*p* = 0.021, T = 2.33), between cerebellar SAL and cerebellar DMN (*p* = 0.016, T = 2.43), and between cortical ECN and cortical SAL (*p* = 0.034, T = 2.13), compared with HCs. However, there was hypoconnectivity between cerebellar DMN and cortical SAL (*p* = 0.040, T = −2.06) among CHR individuals. There was no significant group difference between HCs and URs.

Compared with the CHR group, the FEP group presented hyperconnectivity between cerebellar ECN and cortical SAL (*p* = 0.014, T = 2.50), between cerebellar SAL and cortical SAL (*p* = 0.014, T = 2.50), between cerebellar DMN and cortical SAL (*p* = 0.002, T = 3.11), and between cerebellar SAL and cortical ECN (*p* = 0.012, T = 2.55), whereas hyperconnectivity between cerebellar SAL and cerebellar DMN (*p* < 0.001, T = −3.63) was detected.

### Between-network connectivity: thalamic–cerebellar network connectivity

**Fig. 3:**
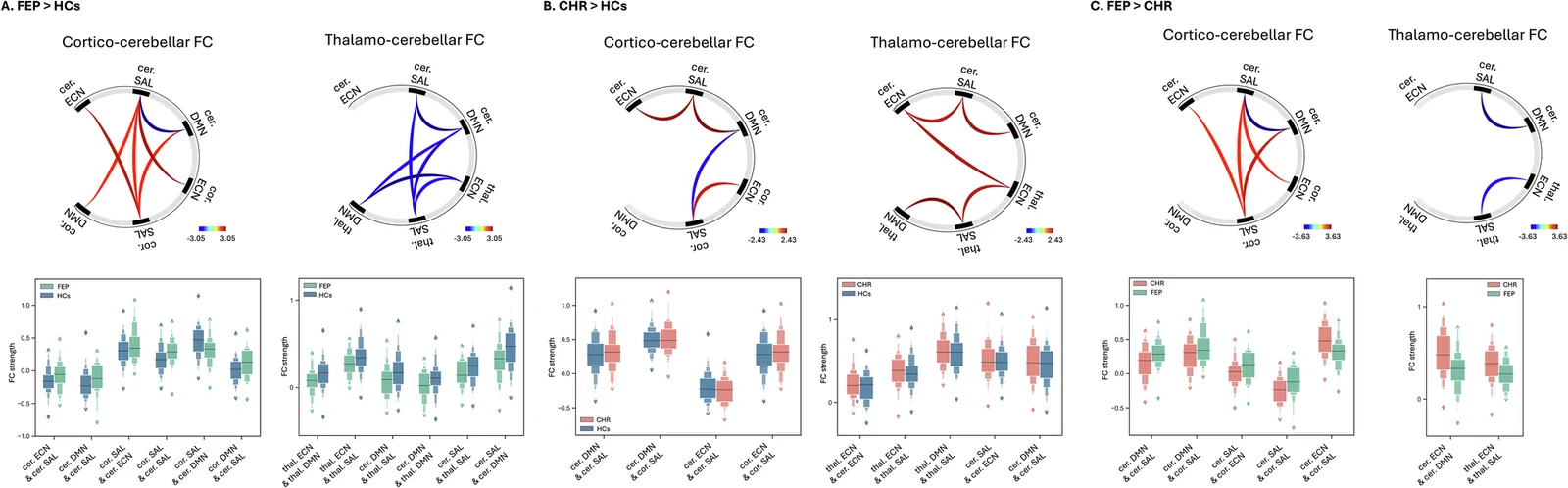
Between-network functional connectivity analysis of cortical‒cerebellar and thalamo-cerebellar circuits. The top is the circular plot, and the bottom is the bar plot representing **A** Comparison between the FEP patients and HCs. **B** Comparison between the CHR individuals and HCs controlling for age as a covariate. **C** Comparison between the FEP patients and the CHR individuals. The URs did not show statistically significant group differences. FC functional connectivity, DMN default mode network, ECN executive central network, SAL salience network, FEP first-episode psychosis, HCs healthy controls, CHR clinical high risk, cer cerebellar, cor cortical, thal thalamic.

Compared with the HC group, the FEP group presented lower connectivity between cerebellar SAL and thalamic SAL (*p* = 0.043, T = −2.04), between cerebellar SAL and cerebellar DMN (*p* = 0.003, T = −3.00), between cerebellar DMN and thalamic DMN (*p* = 0.018, T = −2.42), between thalamic DMN and thalamic ECN (*p* = 0.003, T = −3.05), and between thalamic SAL and thalamic ECN (*p* = 0.017, T = −2.43). Compared with the HCs, the CHR group demonstrated thalamo-cerebellar hyperconnectivity, which was detected between cerebellar ECN and cerebellar SAL (*p* = 0.021, T = 2.33), between cerebellar SAL and cerebellar DMN (*p* = 0.016, T = 2.43), between cerebellar ECN and thalamic ECN (*p* = 0.018, T = 2.38), between thalamic SAL and thalamic ECN (*p* = 0.020, T = 2.35), and between thalamic DMN and thalamic SAL (*p* = 0.010, T = 2.60). Compared with CHR, FEP showed lower connectivity between thalamic ECN and thalamic SAL (*p* = 0.005, T = −2.89) and between cerebellar SAL and cerebellar DMN (*p* = 0.005, T = −3.63). There was no significant difference between HCs and URs in terms of thalamic–cerebellar network connectivity. The results of all the between-network analyses are presented in Table 2 and Fig. 3.

**Table 2:** Group comparisons of cerebellar functional network connectivity.

Functional network connectivity	Groups		FC strengths		
			Cluster *p*-FDR corrected	Connectivity level *p*	T
Cortical-cerebellar network connectivity					
	FEP	HCs			
Cluster A			<0.001^***^		
corDMN_cerSAL	−0.07 ± 0.23	−0.15 ± 0.20		0.041	2.06
corSAL_cerDMN	−0.12 ± 0.23	−0.21 ± 0.21		0.030	2.20
corSAL_cerSAL	0.40 ± 0.27	0.30 ± 0.23		0.038	2.09
corSAL_cerECN	0.29 ± 0.21	0.18 ± 0.21		0.008^*^	2.69
cerDMN_cerSAL	0.32 ± 0.22	0.50 ± 0.24		0.003^*^	−3.00
corECN_cerSAL	0.11 ± 0.23	0.01 ± 0.19		0.006^*^	2.78
	CHR	HCs			
Cluster A			0.033		
corECN_corSAL	0.31 ± 0.26	0.27 ± 0.26		0.034	2.13
cerECN_cerSAL	0.50 ± 0.22	0.49 ± 0.18		0.021	2.33
cerDMN_corSAL	−0.25 ± 0.19	−0.21 ± 0.21		0.040	−2.06
cerDMN_cerSAL	0.31 ± 0.26	0.273 ± 0.26		0.016	2.43
	FEP	CHR			
Cluster A			<0.001^***^		
cerECN_corSAL	0.286 ± 0.207	0.165 ± 0.249		0.014	2.50
cerSAL_corSAL	0.399 ± 0.267	0.269 ± 0.241		0.014	2.50
cerSAL_corECN	0.114 ± 0.227	0.005 ± 0.195		0.012	2.55
cerDMN_corSAL	−0.116 ± 0.234	−0.250 ± 0.191		0.002^**^	3.11
cerDMN_cerSAL	0.315 ± 0.222	0.494 ± 0.247		< 0.001^***^	−3.63
Thalamic-cerebellar network connectivity					
	FEP	HCs			
Cluster A			0.018		
thalECN_thalDMN	0.07 ± 0.15	0.17 ± 0.16		0.003^**^	−3.05
thalECN_thalSAL	0.27 ± 0.15	0.36 ± 0.19		0.017	−2.43
Cluster B			0.018		
cerDMN_thalSAL	0.08 ± 0.19	0.18 ± 0.20		0.008^**^	−2.70
cerDMN_thalDMN	0.02 ± 0.17	0.10 ± 0.17		0.018	−2.41
cerSAL_thalSAL	0.16 ± 0.16	0.23 ± 0.17		0.043	−2.04
Cluster C			0.031		
cerSAL_cerDMN	0.32 ± 0.22	0.45 ± 0.24		0.003^**^	−3.00
	CHR	HCs			
Cluster A			0.009^**^		
thalECN_thalSAL	0.38 ± 0.19	0.36 ± 0.19		0.020	2.35
thalECN_cerECN	0.22 ± 0.18	0.18 ± 0.18		0.018	2.38
thalDMN_thalSAL	0.61 ± 0.21	0.59 ± 0.22		0.010	2.60
cerSAL_cerECN	0.50 ± 0.22	0.49 ± 0.18		0.021	2.33
cerDMN_cerSAL	0.49 ± 0.25	0.45 ± 0.24		0.016	2.43
	FEP	CHR			
Cluster A			0.047		
cerSAL_cerDMN	0.32 ± 0.22	0.49 ± 0.25		0.005^**^	−3.63
Cluster B			0.047		
thalECN_thalSAL	0.27 ± 0.15	0.38 ± 0.19		0.005^**^	−2.89

*cer* cerebellar, *CHR* clinical high-risk, *cor* cortical, *DMN* default mode network, *ECN* executive central network, *FC* functional connectivity, *FDR* false discovery rate, FEP first-episode psychosis, HCs healthy controls, *SAL* salience network, *thal* thalamic.

^**^ *p* < 0.01;^***^ *p* < 0.001.

This table indicates the mean values and the strength of the cerebellar‒cortical functional connectivity that significantly differed between each group on the basis of the cluster-level threshold FDR-corrected *p* < 0.05 and post hoc connection-level threshold *p* < 0.05.

The comparisons between the CHR individuals and the HCs were controlled for age differences.

The data are presented as the mean ± standard deviation.

### Exploratory correlation analysis with error processing in FEP patients

Independent t-test results indicated that the FEP group presented significantly lower performance rates on the error processing task than the HCs, with a highly significant difference (*p* < 0.001, T = −6.48) (Table S2 in the Supplementary Materials). Among the abnormal functional connectivity patterns identified in the FEP group, the connectivity between cerebellar SAL and thalamic SAL showed a statistically significant negative correlation with task error rates (r = −0.47, uncorrected *p* = 0.003, FDR-corrected *p* = 0.041; Supplementary Figure S1 and Supplementary Table S1).”

## Discussion

This study examined cerebellar–thalamic and cerebellar–cortical connectivity networks across familial risk, CHR, and FEP using the triple network model as an organizing framework, and demonstrated group-specific patterns rather than a uniform shift in CTC pathways. Our results showed widespread cross-regional disruptions in cerebellar–thalamic and cerebellar–cortical pathways in FEP patients. In contrast, CHR individuals exhibited more localized alterations within cerebellar, thalamic, and cortical networks, whereas cerebellar connectivity remained intact in URs. By systematically evaluating these patterns, the present study advances understanding of large-scale CTC network involvement and highlights the potential role of cerebellar connectivity in early psychosis.

**Fig. 4:**
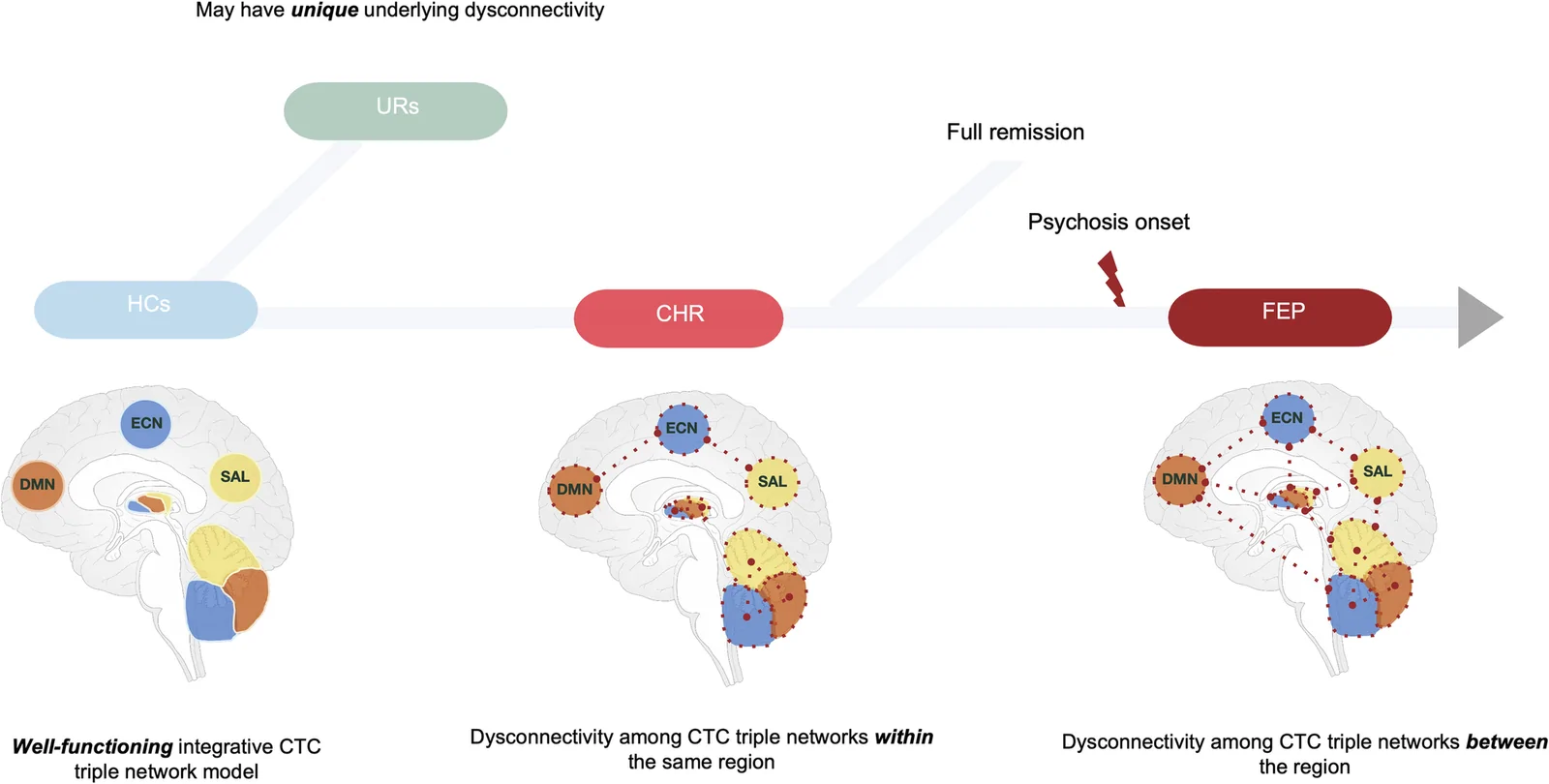
Summary of the functional connectivity results. The findings revealed abnormal cerebellar connectivity within the CTC circuit, varying by psychosis risk level and early stage, and reflected distinct disruptions in the large-scale triple network model. FEP first-episode psychosis, HCs healthy controls, CHR clinical high-risk, URs unaffected relatives, DMN default mode network, ECN executive central network, SAL salience network, CTC cortico-thalamo-cerebellar.

In FEP patients, we observed increased cerebellar–cortical connectivity alongside reduced cerebellar–thalamic connectivity. This pattern aligns with previous reports showing thalamo–cerebellar hypoconnectivity within the DMN and ECN^15^ and thalamo–cortical hyperconnectivity^33^ in FEP patients. These findings indicate that different segments of the CTC system may be affected in distinct ways. Although mechanistic interpretations cannot be drawn from resting-state data, the coexistence of increased and decreased connectivity within interconnected pathways echoes interpretations in prior work that such heterogeneity can emerge when broader network organization is disrupted. Rather than a uniform shift in one direction, these findings point to a more complex pattern of CTC alteration in FEP, in which multiple circuits appear to change in different ways. Viewed together with earlier study of chronic schizophrenia^32^, the present results indicate that deviations across the CTC system are already identifiable at the early stage of psychosis and may reflect the initial configuration of large-scale network disturbance (Fig. 4). The increased cerebellar–cortical connectivity we observed in FEP accords with a recent synthesis of antipsychotic-naïve first-episode schizophrenia, in which cerebello-cortical alterations were among the most consistently reported functional changes, including increased connectivity in patients and their unaffected siblings and more pronounced changes in at-risk individuals who later converted to psychosis^43^. A meta-analysis of cognitive task fMRI similarly places cerebellar dysfunction within the broader cortico-striato-thalamo-cerebellar circuit linked to working memory and cognitive control^44^. These convergent findings suggest that the cerebellar–cortical increase reflects a reconfiguration of the cerebellum within disrupted large-scale circuits rather than an isolated regional change, though whether it is compensatory or maladaptive cannot be resolved with the present cross-sectional data^45^.

Previous studies have reported abnormal cerebellar connectivity to cortical clusters related to the triple network model among CHR individuals^10,11^. Kim et al.^33^ reported increased cortical DMN-thalamic SAL connectivity, increased cortical DMN-thalamic DMN connectivity, and decreased cortical DMN-SAL connectivity, which suggests that cortical–thalamic triple network disturbances may precede more widespread alterations. In the present study, CHR participants showed localized cerebellar-related disruptions, contrasting with the more extensive cross-regional disturbances observed in FEP. This distinction aligns with accounts suggesting that dysconnectivity in psychosis may emerge in more circumscribed regions prior to broader involvement across interconnected networks^46^.

Interestingly, URs did not show significant cerebellar connectivity differences compared with HCs, contrasting with some prior studies on familial risk for psychosis. For example, Repovs et al.^19^ reported abnormal cerebellar connectivity with SAL and ECN cortical networks in siblings of patients, whereas Collin et al.^18^ found no significant differences. Such inconsistencies likely reflect differences in risk definitions or cohort characteristics. In our study, as in Kim et al.^33^, URs were required to have a first-degree relative with schizophrenia and additional affected relatives up to the third degree, while remaining free of psychiatric symptoms, thereby representing a group enriched for familial loading despite the absence of clinical symptoms. Under these criteria, Kim et al.^33^ observed thalamic ECN hypoconnectivity with cortical DMN and SAL networks in URs, whereas our analysis did not identify significant cerebellar abnormalities. Within this framework, the preserved cerebellar connectivity observed here may suggest that CTC interactions involving the cerebellum remain relatively intact in asymptomatic individuals, whereas the disruptions detected in CHR and FEP may be more closely associated with emerging clinical features. This interpretation is tentative and should be examined in studies incorporating genetic stratification; nevertheless, overall, these findings raise the possibility that cerebellar triple network alterations may relate more closely to symptom expression than to familial risk alone.

In both FEP patients and CHR individuals, we found alterations in cerebellar–thalamic and cerebellar–cortical connectivity mostly involving the SAL. Given the SAL directs transitions between the DMN and the ECN, disturbances in this system can interfere with the ability to judge which information is relevant^47^, and psychosis often involves this form of altered salience processing^48,49^. Notably, prior neuroimaging studies have consistently shown abnormal SAL engagement during error-related tasks in psychosis and at-risk groups^50–52^, and have suggested that salience-related processes may be affected early in the illness course. With the emerging evidence suggesting the influence of the cerebellum and thalamus in cognitive control and modulation of cortical regions^29,53–56^, it can be inferred that the cerebellum and thalamus may contribute to this process by influencing how information is integrated and relayed to cortical systems involved in salience detection and cognitive control (Supplementary Figure S2).

In this context, our exploratory observation that lower cerebellar–thalamic SAL connectivity was associated with greater error rates during the SWM in FEP provides a preliminary indication of possible behavioral relevance. Compared with CHR individuals, the more pronounced SAL-related connectivity alterations observed in FEP may suggest that disruption of this circuit becomes more evident as clinical symptoms emerge, whereas the CHR group may exhibit more localized or heterogeneous alterations at an earlier stage. Although the nature of this relationship cannot be clearly determined from the current data, the convergence between our connectivity findings and prior evidence of SAL dysfunction, and anatomical accounts of cerebellar influence on cortical coordination via thalamic pathways^53–56^ suggests that cerebellar–thalamic interactions may be involved in salience-related processes in early psychosis.

These observations raise the possibility that alterations in SAL-related CTC circuits could contribute to cognitive difficulties in psychosis, and motivate further work designed to directly test these mechanisms^57^. In contrast to the robust group-level CTC connectivity alterations, the association with behavioral phenotype was more modest and should therefore be interpreted with caution. In the present study, phenotype analysis was limited to an exploratory examination of the relationship between connectivity strength and SWM error-processing performance in the FEP group, and associations with specific clinical symptom dimensions were not examined. One possible explanation is that CTC dysconnectivity reflects disturbances in large-scale circuit coordination rather than a direct linear marker of symptom or cognitive severity. The CTC system has been implicated in distributed processes including prediction, error monitoring, and cognitive coordination^20–23^, and alterations in this circuitry may therefore contribute to general psychosis-related dysfunction at a systems level rather than mapping onto a single behavioral measure. This interpretation suggests that clear group differences across illness stages may coexist with more modest brain–behavior associations within a given clinical group.

Our study has several limitations. First, we did not perform analyses to investigate topological dynamics. While our functional connectivity findings demonstrate group differences in connectivity strength, the abnormal network dynamics associated with psychosis, particularly in terms of the integration and segregation of information within and between networks, remain an area for further exploration^58^. Future research could extend this work by examining the dynamics of CTC triple networks to elucidate these processes in greater detail. Second, the URs were not stratified by polygenic risk scores. Although our inclusion criteria ensured the selection of asymptomatic individuals, this approach may have constrained our ability to comprehensively assess genetic contributions to network disruptions. Third, the effect of handedness should be considered when interpreting our results. Although it did not differ across groups in the present sample, handedness has been highlighted as a potentially relevant factor in neuroimaging studies^59^. Given the consistency across our previous studies and to preserve correspondence with the network parcellation maps derived from the full sample, we did not exclude participants based on handedness. Its potential influence nevertheless cannot be entirely disregarded, and future studies with larger samples that explicitly account for handedness would help clarify this point. Lastly, we did not evaluate functional connectivity in CHR individuals who transitioned to psychosis due to the limited sample size; therefore, the findings should be interpreted with caution. A comparative analysis of those who transition to psychosis versus those who remit from the CHR group would offer critical validation of our hypotheses. Future studies employing longitudinal designs would be particularly valuable in addressing these limitations and delineating connectivity changes over the course of psychosis progression.

In conclusion, the present study addresses a critical gap in the understanding of how cerebellar interactions contribute to large-scale network disruptions within the CTC triple network model across levels of psychosis risk and stage. Our findings provide a descriptive outline of how CTC circuits diverge across early stages of psychosis and indicate that cerebellar-related network alterations may emerge in parallel with changes discussed in the triple network model. Considering the results within this framework helps situate cerebellar and thalamic pathways within broader discussions of early network involvement in psychosis. Together, these observations offer a refined characterization of early CTC involvement and provide a basis for future studies aimed at clarifying their relevance for psychosis risk and illness onset.

## Supplementary information

Supplementary information

## Data Availability

The data that support the results of this study are available from the corresponding author upon reasonable request. The data are not available because they contain information that could compromise the privacy of the research participants.
