# Supplementary material for: Disrupted cerebellar interactions in the cerebellar-thalamo-cortical triple network model across risk states and first-episode psychosis
Source: Schizophrenia (Heidelb). 2026 Jul 16;12(1):87. doi: 10.1038/s41537-026-00783-6 (PMC13745458; doi:10.1038/s41537-026-00783-6)
Supplement: Supplementary file 1 [file 41537_2026_783_MOESM1_ESM.docx]

**SUPPLEMENTARY MATERIALS**

**Figure S1.** Regression plot of correlations between the SWM task performance and connectivity strength between the cerebellar and thalamic SAL networks in FEP patients.

**Figure S2.** Expected cortico-thalamo-cerebellar triple network model.

**Table S1**. Correlation analysis between network functional connectivity and error rates in FEP patients.

**Table S2.** Group differences between FEP patients and HCs in performance on the spatial working memory task.

**Figure S1.** Regression plot of correlations between the SWM task performance and connectivity strength between the cerebellar and thalamic SAL networks in FEP patients.

The y-axis representing the error rates of the task and the x-axis representing the connectivity strength between the cerebellar SAL and thalamic SAL in FEP patients.

FC, functional connectivity; SAL, salience network; FEP, first-episode psychosis; HCs, healthy controls; SWM, spatial working memory task

**Figure S2.** Expected cortico-thalamo-cerebellar triple network model. Within this framework, we carefully suggest that disrupted salience mapping may result from perturbed cortico-cerebellar connectivity, in which cerebellar networks, possibly regions related to the SAL, function as the core processor. Thus, impaired cerebellar-thalamic connectivity could hinder the proper coordination of the commands, whereas disruptions in thalamo-cortical connectivity may affect the integration of new information, exacerbating cognitive and perceptual disturbances.

**Table S1**. Correlation analysis between network functional connectivity and error rates in FEP patients.

| **Functional network connectivity** | **Statistics** | | |
| --- | --- | --- | --- |
|  | ***r*** | ***p*** | **FDR-corrected *p*** |
| thalECN_thalDMN | -0.20 | 0.289 | 0.694 |
| thalECN_thalSAL | -0.21 | 0.196 | 0.694 |
| cerDMN_thalSAL | -0.30 | 0.182 | 0.694 |
| cerDMN_thalDMN | 0.12 | 0.742 | 0.809 |
| cerSAL_thalSAL | -0.47 | 0.003^**^ | 0.041^*^ |
| cerSAL_cerDMN | -0.23 | 0.695 | 0.809 |
| corECN_cerSAL | 0.14 | 0.705 | 0.809 |
| cerDMN_cerSAL | -0.23 | 0.695 | 0.809 |
| corSAL_cerECN | -0.13 | 0.574 | 0.809 |
| corSAL_cerSAL | 0.10 | 0.488 | 0.809 |
| corSAL_cerDMN | -0.12 | 0.942 | 0.942 |
| corDMN_cerSAL | 0.18 | 0.255 | 0.694 |

This table shows the results of the correlation analysis between functional connectivity values that were found to be significant from the group comparison compared with the HCs and the error rates of spatial working memory in FEP patients.

FEP, first-episode psychosis; DMN, default mode network; ECN, executive central network; SAL, salience network; FDR, false discovery rate; cer, cerebellar; thal, thalamic; cor, cortical

^*^p < 0.05; ^**^p < 0.01; ^***^p <0.001

|  | Groups | | Statistics | |
| --- | --- | --- | --- | --- |
|  | FEP | HCs | *p* | T |
| Accuracy rates | 11.23 ± 10.61 | 35.20 ± 21.47 | <0.001** | -6.48 |

**Table S2.** Group differences between FEP patients and HCs in performance on the spatial working memory task.

Independent samples t-tests were conducted, and the data are presented as the means ± standard deviations.

FEP, first-episode psychosis; HCs, healthy controls.

^***^p <0.001.
